# Evaluation of performance-enhancing drugs seized by Israeli enforcement agencies 2012–2017: implications for policy and regulatory change

**DOI:** 10.1186/s13584-020-00369-2

**Published:** 2020-05-04

**Authors:** Hagit Bonny-Noach, Ronny Berkovitz, Barak Shapira

**Affiliations:** 1 The Department of Criminology, School of Social Sciences, Ariel University, Ariel, Israel; 2 Israeli society of Addiction Medicine (ILSAM), Ramat-Gan, Israel; 3 Israel National Anti-Doping Organization (ISR-NADO), Tel-Aviv, Israel; 4 Division of Enforcement and Inspection, Israel Ministry of Health, Jerusalem, Israel

**Keywords:** Doping, Seizures, Performance-enhancing substances, Androgenic anabolic steroids, Enforcement, Israel

## Abstract

**Background:**

Illicit performance-enhancing substances are used mostly by athletes to enhance performance in sports, and by bodybuilders to gain muscle and body mass. Among performance-enhancing substances, the most common and known substances are anabolic-androgenic steroids, which are associated with a range of short and long-term adverse medical and psychiatric effects.

While the sale and distribution of performance-enhancing substances are considered criminal offenses per the Israeli local pharmacy ordinance, the use and personal possession of these substances are not. Presently, the Division of Enforcement and Inspection of the Israel Ministry of Health cooperates with police and customs agents in performance-enhancing substance-related enforcement activities, which chiefly include seizures carried out at suspicious sites. Moreover, the Division of Enforcement and Inspection provides professional guidance, lab analysis services, and expert opinions on the toxicological and pharmaceutical nature of products seized.

This paper presents a contemporary sub-analysis of registered seizures of performance-enhancing substances carried-out by Israel enforcement agencies. The main aim of this analysis is to characterize current patterns of performance-enhancing substances, thus providing the possibility of better assessment of current enforcement and health policy.

**Methods:**

A sub-analysis of 712 seizures of performance-enhancing substances seized by Israeli enforcement authorities during a six-year period ranging from January 2012 to December 2017.

**Results:**

This study demonstrates that Israel faces a challenge regarding the importation and distribution of illicit performance-enhancing substances. The most common substances seized were anabolic androgenic steroids (*N* = 539). Most seizures were carried out in the central mail processing sites, (38.4%), followed by seizures in private premises such as homes and warehouses (29.6%). Significant differences were found between anabolic-androgenic steroids and other substances, relating to place and year of seizure. Among seizures with known sources (*N* = 355), the most frequent geographic region given as the source of substances was Eastern Europe (47.6%), followed by East Asia (24.8%), West Asia (19.4%), and Western Europe (5.9%). Bulgaria was the country with the highest frequency of seizures (*N* = 71) followed by Jordan (*N* = 45), Thailand (*N* = 37) and Moldova (*N* = 36). Significant regional differences were found based on the variables of gender, place of seizure, and type of substance. The most frequent month of seizures was August (*N* = 129), followed by July (*N* = 119), and June (*N* = 118).

**Conclusions:**

While data analysis focused on the supply side of the performance-enhancing substances market, the high number of seizures of performance-enhancing substances in Israel represents evidence of the existence of a high demand and a large consumer base for these products. Consequently, there is a need for developing further enforcement, treatment, and prevention policies that do not currently exist in Israel. Policymakers should consider prioritizing law enforcement action and incentivizing intelligence sharing to monitor suspected shipment sources and specific points of entry. Additionally, the results demonstrate that there is a need in reforming the penal law to discourage the use of performance-enhancing substances. Similar measures have already been applied in countries like Spain, Italy, and Belgium. Furthermore, policy-makers should consider enhancing health ministry agencies with a higher enforcement capacity by giving them further investigative and inquiry authority. Due to the troubling magnitude of the phenomenon, policymakers should also prioritize educational and prevention strategies.

## Introduction

The terms performance-enhancing supplements, performance-enhancing substances (PES) as well as performance and image-enhancing drugs (PIEDs) are used interchangeably to describe substances which are illicitly employed by athletes to enhance performance in sports, and by bodybuilders to gain muscle and body mass [1]. Anabolic-androgenic steroids (AAS) are some of the most common PES identified in anti-doping tests [2]. Moreover, the worldwide trade in AAS has increased significantly since the late 2000s [3]. PES are associated with a range of short and long-term adverse medical and psychiatric effects, violent behavior and suicide [3–8].

In Israel, the sale and distribution of AAS are considered criminal offenses, yet, the prohibition of possession and use is not enforced. Only approved AAS may be sold and under several limitations. By law, AAS are defined as prescription-only drugs and may be sold only by a pharmacist upon the presentation of a medical doctor’s prescription. Accordingly, the sale, production, importation, and exportation of performance-enhancing substances are illegal and could lead to 3 years in prison [9]. Enforcement of the law is mainly under the responsibility of the Israeli police. The Ministry of Health Division of Enforcement and Inspection (DEI) is responsible for providing professional guidance to enforcement agencies, providing lab analysis services and expert opinions on the toxicological and pharmaceutical nature of products seized. The DEI accompanies police and customs agents in enforcement activities, including searches, raids, and seizures carried out at suspicious sites. Despite ongoing enforcement activities, most cases involving seizure of PES are still carried out by the Israeli customs service, which prioritizes revenue and tax collection rather than the enforcement of the criminal code regarding PES trafficking. Additionally, the fight against PES is not currently prioritized by the Israeli police. Thus, PES production and procurement are seldom prosecuted, and sentencing is usually minor for producers and procurers.

Despite the growing evidence of a thriving PES black market, there is a lack of data regarding the prevalence and incidence of PES use in Israel. The Israel Anti-Drug Authority (IADA) and its successor, the Israel Anti-Drug, Alcohol and Violence Authority (IADAV), which collects data on substance abuse, does not include PES use in its annual epidemiological study [10]. There are currently only two studies about AAS use among the population of Israel. One study, published in 2006 reported on the consumption of AAS among a sample of 550 young students at an academic center, comparing students from five European Union countries. It found that only 1.4% (*N* = 6) of Israelis reported doping [11]. The second study was on the consumption of AAS among a sample of 665 amateur athletes attending a gym. In this study, 4.6% reported using anabolic steroids, 21.3% reported that they had been offered to use steroids, 48.9% reported that they knew a person who uses steroids, and 22.8% reported having friends who use steroids [12].

While the pharmacist ordinance governs all provisions regarding the importation, procurement, and sale of pharmaceuticals, in general, it is the Israel Sports Law, which establishes the prohibition of their use in sports. This law, which is under the responsibility of the Ministry of Culture and Sports, allows the Minister of Sports, with the consent of the Minister of Health, to declare by decree some drugs and stimulants prohibited for use under a specific subsection. Additionally, it allows for the installment of procedural rules governing tests to identify doping agents, allows for the locating of athletes who have violated anti-doping rules, and makes testing for athletes mandatory. Violation of the provisions of this law is considered a disciplinary offense. Nevertheless, it should be noted that this law does not govern activities in non-federated sports such as bodybuilding and wrestling. Thus, it only serves to maintain “fair-play” within sports and does not deal with trafficking, procurement, and distribution of PES [13].

In December 2011, the government of Israel joined the United Nations Educational, Scientific and Cultural Organization (UNESCO) Convention against doping in sports [14]. In order to comply with the convention principles, the Israeli Committee for the Prevention of Doping, established by the Israel Olympic Committee in 1991, was re-organized as the Israel National Anti-Doping Organization (INADO)*.* In Israel, INADO is the local representative of the World Anti-Doping Agency (WADA), and although it is an independent body, it is the highest local authority in the prevention of doping in sports. Information regarding actual use of PES and AAS in particular by athletes of all disciplines is incomplete, as this data is only available for professional athletes in associated or federated sport disciplines. In 2014, only one out of 160 tests were found positive for PES use. In 2015, out of 131 tests conducted, none returned positive results. In 2016, three out of 344 tests carried reported positive results [15]. The WADA Code encourages state authorities to collaborate with sports federations and associations [16]. In the spirit of the above code, there is a burgeoning collaboration between the DEI and INADO. This cooperation is particularly visible in the areas of information sharing and the drafting of a comprehensive Israeli anti-doping law. Nevertheless, it appears that most activities relating to PES procurement and distribution are carried-out outside professional sports, and within circles of body-builders and gym frequenters.

Most of the studies relating to AAS are from the medical and psychology literature, as well as from studies exploring the sociology of sport, health, and masculinity [17]. Hence, the use of AAS has been chiefly explored from a user-perspective [1, 18, 19]. However, analysis of the supply-side of the market for doping products has been widely neglected [3, 17, 20]. Consequently, very little has been published providing a statistical analysis of law enforcement seizures of PES, although some authorities do publish partial or full reports on seizures within their jurisdictions, as presented in Table 1.

**Table 1:** Comparison of periodic seizures of performance-enhancing drugs (PED) in other jurisdictions

Country	Period	Quantity seized (kg or doses)	Top dispatcher countries indicated in current or previous reports	Additional information
Australia [21]	2017–2018	75.7 kg	United States, United Kingdom, China, Hong Kong, Thailand, Turkey, India	72.3% of seizures were of Anabolic-androgenic steroids, 27.7% other PEDs
England and Wales [22]	2017–2018	1,700,000 doses	China, India, Pakistan [23, 24]	
Germany	2017–2018	750,000 doses [25],	China, India, United States, Canada [26–28]	
Switzerland [29, 30]	2013–2014	264,631 doses	Greece, Slovakia, China, United States	74% of PED seizures: Anabolic-androgenic steroids
United States [31, 32]	September 2015	134,000 doses 636 kg. bulk material, 8200 l of injectable steroids	Mexico, China. Romania, Greece,	Operation “Cyber-Juice”

Additionally, recent literature, mostly from Western European countries, focuses on consumers of PES [33], such as those from Switzerland [29, 30], Italy [3], and the United Kingdom [34, 35]. Nevertheless, when the source of AAS is discussed in these works, it is consistently reported as primarily manufactured in Asian countries, but shipped to Western European nations via South-Eastern Europe [29, 30]. AAS is reported to be frequently produced in pharmaceutical laboratories in countries with lax or favorable regulation, or in small underground laboratories where stricter laws are present [17]. The macro-area of East Asia is known as a major source of worldwide doping agents, particularly Thailand, China, and India, with numerous production sites and well-established trading routes. In contrast, Eastern European countries, in particular, Ukraine, Lithuania, Romania, Poland, Hungary, and Slovenia are mainly distribution hubs, thus controlling a considerable share of the illegal international market for PES [33].

This study aims to report PES seizures carried-out by Israeli enforcement agencies (The Ministry of Health Division of Enforcement and Inspection (DEI), police and customs agents), in order to uncover meaningful patterns regarding PES trafficking and procurement. As such, we examined possible associations between geographical region, location of seizures, the type of substance seized, and suspects’ gender. In general, information regarding PES trafficking and use may come from a variety of sources, which include statistics provided by law enforcement authorities [36]. Currently, the Israeli PES market remains largely under-researched. Consequently, a report on PES trends and patterns could provide readers with a better understanding of the characteristics of the Israeli PES market, and help policy-makers in formulating legal and practical responses to this emerging public health risk.

## Methods

### Data collection

A survey was carried out of all pharmaceutical crime cases recorded in the DEI electronic database (Barillet Software Solutions, Bnei Brak) between January 1st 2012 and 31st December 2017. The database is digital and stored in the Ministry of Health intranet system. Information on pharmaceutical crime cases are manually added by DEI employees for case follow-up and report. The survey yielded a total of 5119 cases. Among all these cases, we selected 712 cases, which included reports of seizures of performance-enhancing drugs. Information regarding the identity of senders, suspects, personal information, and the identities of the investigators and officers involved was omitted from the analysis.

### Inclusion criteria

Inclusion criteria: 1) all cases in which a WADA prohibited substance was involved, and 2) Known doping substance used by bodybuilders. Cases were selected by a pharmacist working in the DEI, with knowledge of the database. The following data were extracted from each case, and sorted according to the following categories: Type of substance, commercial name of substance, confirmed identity of substance (where lab analysis was available), minimum quantity confirmed as seized, place of seizure, product presentation (vial, tablet or raw powder), last known origin country of product (when reported) and the gender of the involved suspect. Where cases did not contain data about these categories, we merely categorized them as “unknown.”

### Sample collection

All 712 cases involved seizure of performance-enhancing drugs by police, customs, and health ministry inspectors. Samples of seized substances were transferred to DEI pharmacists for visual inspection, or lab analysis, to determine their nature and content. Upon receiving a sample, DEI personnel recorded all relevant information in the database regarding the seizure, place of seizure, circumstances of seizure, and suspects’ name and characteristics. If samples lacked labels or descriptions, DEI sent them for lab analysis to one of the two forensic laboratories in Israel: The Sheba toxicological lab and the Ministry of Health analytical laboratories. These labs are authorized to process evidence, and their expert opinions are admissible as legal testimony.

### Descriptive and quantitative data sub-analysis

Data were analyzed using SPSS 21 (IBM, Armonk).

We provided descriptive statistics of the following variables: Year and month of seizures; source country and region - if disclosed previously by customs and police; sex of recipient or of the last known holder of the seized products; and minimum quantity seized - denoted by number of dosing units or potential dosing units if the seized product was bulk product. We performed a chi-square test to examine differences between variables, with the aim to document variations in seizures of anabolic steroids and other PES by gender, year of seizure, and place of seizure. The “Others” group of PES in the analysis was comprised of the following categories: Adrenergic and sympathomimetic agents (e.g. ephedrine, clenbuterol); diuretics, sodium and potassium supplements (e.g. furosemide, potassium chloride) hormones, growth hormones, and hormone releasing agents (e.g. somatropin, liothyronine, FSH, menotropin); post-cycle and adverse effect treatment agents (e.g. anastrazole, tamoxifen, clomifene citrate); and site-enhancing oils (“synthol”).

## Results

**Fig. 1:**
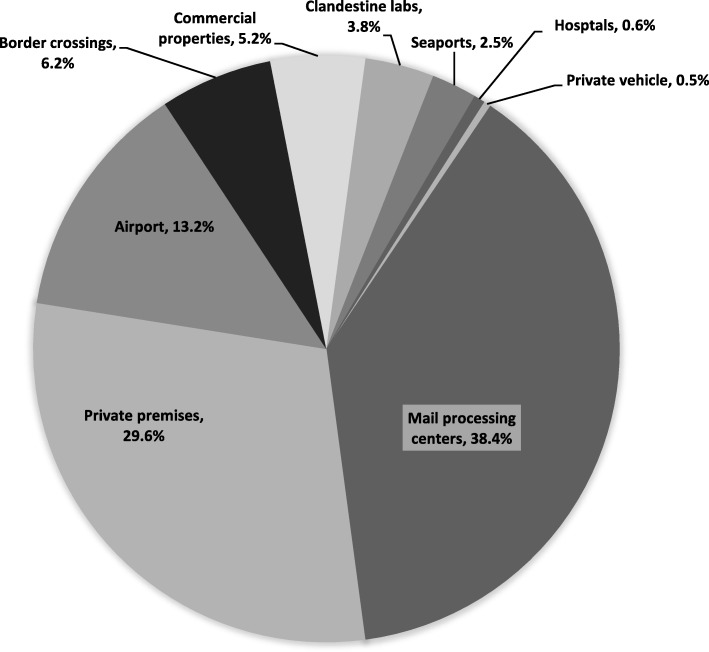
Place of seizure by percentage of total seizures (2012–2017) (*N* = 712)

During the 6 year period under review (January 2012 to December 2017), there were 714 seizures of suspected PES carried out by Israeli authorities. In sum, 712 cases were recorded as confirmed PES seizures, while two were later categorized as unrelated (both were later confirmed as seizures of sildenafil analogs). The locations where most seizures were carried out were mail processing centers located in Tel-Aviv, Haifa and Modi’in, which distribute packages to local post-offices (37.8%), followed by private premises such as homes, shops and private storage sites (29.6%). Other seizures were carried out at the Ben-Gurion Tel-Aviv airport (13.2%) and the two border crossings to Jordan – Allenby Bridge and the Jordan River (6.2%), commercial properties, comprised of shops, stores, and gyms (5.2%), clandestine domestic PED labs across Israel (3.8%), the seaports of Eilat and Ashdod (2.5%), hospitals (0.6%), or from private vehicles such as cars and motorcycles (0.4%) (Fig. 1).

**Fig. 2:**
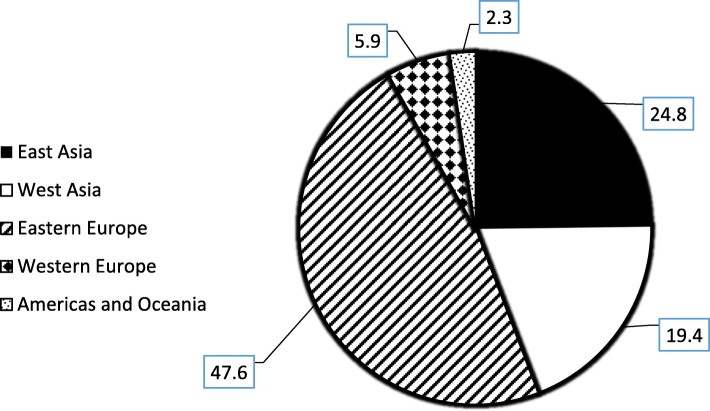
Percentage of all disclosed seizures cases by geographical region (last known) (2012–2017) (*N* = 355)

For over half of the seizures (*N* = 357), the source of seized substance was unknown or was not revealed by the suspects. For those seizures in which the substance source was revealed (*N* = 355), the most frequent geographic region identified as the immediate source of the substance was Eastern Europe (47.6%), followed by East Asia (24.8%), West Asia (19.4%), and Western Europe (5.9%). A small number of seizures (2.3%) was reported to originate from the Americas and Oceania (Fig. 2).

Among seizure cases with a known source, the country with the highest incidence rate of seizures was Bulgaria (*N* = 71), followed by Jordan (*N* = 45), Thailand (*N* = 37) and Moldova (*N* = 36). The total confirmed seized quantity represents the quantity of raw powder confirmed as seized in the system. The source country with the highest total minimum confirmed seized quantity was Thailand (65,011 dosing units), followed by Moldova (29,125 dosing units), Jordan (21,857 dosing units) and Bulgaria (12,772 dosing units) (Table 2).

**Table 2:** Source country (last known) of products seized and percentage of total seizures (2012–2017) (*N* = 712)

Source country	Number of seizures	Percentage of total seizures
Bulgaria	71	10
Jordan	45	6.3
Thailand	37	5.3
Moldova	36	5.1
Hong Kong	25	3.5
Israel	19	2.8
Belarus	18	2.5
China	15	2.2
Lithuania	14	2.0
Ukraine	13	1.8
India	10	1.4
Poland	10	1.4
U.K	9	1.3
Germany	6	0.8
Turkey	5	0.7
Belgium	3	0.4
Ecuador	3	0.4
Malta	3	0.4
Russia	3	0.4
Australia	2	0.3
United States	2	0.3
Cambodia	1	0.1
Canada	1	0.1
Czech Republic	1	0.1
Hungary	1	0.1
Latvia	1	0.1
Romania	1	0.1
Unknown/Undisclosed	357	50.1
Total	712	100

Regional differences were found in the sub-analysis of 355 seizures from known sources regarding the ultimate known source of substances, defined as the last country reported before entry to Israel. Moreover, as shown in Table 3, regional differences were found based on the variable of gender (χ2 = 25.12, df = 4, *p* < .0005). Most of the cases in which women were involved reported the substance source as Eastern Europe (22.2%), followed by East Asia (4.9%) and West Asia (4.6%). Regional differences were also found regarding the yearly incidence of seizures over time from 2012 to June 2017 (χ2 = 124.20, df = 20, *p* < 0.005). The largest proportion of seizures from East-Asia occurred in 2016 (24.7%). For West Asia the largest proportion of seizures occurred in 2013 (36,9%). Most seizures from Western Europe occurred in 2012 (35.0%) and for Oceania most were registered in 2017 (50.0%). Regional differences were also found with respect to place of seizure (χ2 = 163.64, df = 12, *p* < 0.005). Most of the seizures from East Asian countries were registered in mail and package processing centers (63.0%), while those from the West Asia region were mainly seized at airports and land-ports (63.1%), with a large proportion coming from Jordan. Seizures from Eastern European countries were chiefly seized in mail and packages processing centers (91.2%), as well as those arriving from Western Europe (85.0%) and Oceania (100.0%). Also, regional differences were found regarding the type of substances seized (χ2 = 127.63, df = 20, *p* < 0.005). The largest proportion of adrenergic and sympathomimetic agents arrived from East Asia (4.9%), whereas the largest proportion of AAS was among seizures sourced from East Asia (85.2%) and Eastern Europe (84.2). Seizures from West Asia had the largest proportion of growth hormones and hormone releasing agents (10.8%).

**Table 3:** Case attributes by region (*N* = 332)

Zone	Variable	East Asia*		West Asia^a^		Eastern Europe^b^		Western Europe^c^		Americas and Oceania^d^		df	χ^2^	Sig.
		N	%	N	%	N	%	N	%	N	%			Sig.
		81	24.4	65	19.6	158	47.6	20	6.0	8	2.4			
**Gender**												4	25.12	
	**Male**	77	95.1	62	95.4	123	77.8	20	100.0	8	100			<.0005
	**Female**	3	4.9	3	4.6	35	22.2	0	0.0	0	–			
**Year**												20	124.40	
	**2012**	14	17.3	1	1.5	2	1.3	7	35.0	0	0.0			<.0005
	**2013**	1	1.2	24	36.9	22	13.9	5	25.0	0	0.0			
	**2014**	15	18.5	16	24.6	16	10.1	3	15.0	1	12.5			
	**2015**	16	19.8	12	18.5	16	10.1	0	0.0	1	12.5			
	**2016**	20	24.7	4	6.2	65	41.2	0	0.0	2	25.0			
	**2017**	15	18.5	8	12.3	37	23.4	5	25.0	4	50.0			
**Place of Seizure**												12	163.64	
	**Land and Air ports of entry**	26	32.1	41	63.1	13	8.3	0	0.0	0	0.0			<.0005
	**Mail and Packages processing centers**	51	63.0	6	9.2	144	91.2	17	85.0	8	100.0			
	**Private premises**	3	3.7	12	18.5	1	0.6	3	15.0	0	0.0			
	**Public or commercial properties**	1	1.2	6	9.2	0	0.0	0	0.0	0	0.0			
**Type of substance**														
	**Adrenergic and sympathomimetic agents**	4	4.9	1	1.5	11	3.3	5	1.5	0	0.0			
	**Anabolic Steroids**	69	85.2	49	75.4	133	84.2	13	65.0	5	62.5			
	**Diuretics, Sodium and potassium supplements**	0	0.0	2	3.1	0	0.0	0	0.0	0	0.0	20	127.63	
	**Hormones, Growth hormones, and hormone releasing agents**	7	8.6	7	10.8	5	3.2	1	5.0	0	0.0			<.0005
	**Post-cycle and adverse effects treatment agents**	0	0.0	6	9.2	9	5.7	1	5.0	0	0.0			
	**Site enhancing oils**	1	1.2	0	0.0	0	0.0	0	0.0	3	37.5			

“*” = ^a^East Asia: Cambodia, China, Hong Kong, India, Thailand

^b^West Asia: Israel, Jordan, Turkey

^c^East Europe

^d^West Europe

^e^Americas and Oceania:

The most common substances seized were anabolic androgenic steroids (*N* = 539). As shown in Table 4, significant differences were found between AAS and other substances regarding place (χ2 = 13.59, df = 3, *p* < 0.04) and year of seizure (χ2 = 19.70, df = 5, *p* < 0.01), as most of the AAS seizures were made in 2014 (*N* = 158) (29.3%), followed by 2016 (*N* = 112,20.8%). Seizures of “Other Substances” were carried out mainly in 2016 (*N* = 39,31.2%), followed by 2014 (*N* = 24, 19.2%). AAS were mostly seized in mail processing centers (38.4%) and suspects’ private premises (33.6%), followed by seizures made at land and air points of entry (21.9%). Other substances were mostly seized from private premises (44.0%) and mail processing centers (40.8%).

**Table 4:** Case attributes by type of substances (*N* = 664)

Type	AAS^a^ (*N* = 539)	Others Substances^b^ (*N* = 125)	df	χ ^2^	Sig
	N (%)	N (%)			
**Gender**					
**Male**	489 (90.7)	115 (92)	1	.201	.065
**Female**	50 (9.3)	10 (8.0)			
**Year**					
**2012**	22 (4.1)	7 (5.6)	5	19.701	.001
**2013**	59 (11,0)	23 (18.4)			
**2014**	158 (29.3)	24 (19.2)			
**2015**	87 (16.1)	21 (16.8)			
**2016**	112 (20.8)	39 (31.2)			
**2017**	101 (18.7)	11 (8.8)			
**Place of Seizure**					
**Land and Air ports of entry**	118 (21.9)	10 (8.0)	3	13.594	.004
**Mail and Packages processing centers**	207 (38.4)	51 (40.8)			
**Private premises**	181 (33.6)	55 (44.0)			
**Public or commercial properties**	33 (6.1)	9 (7.2)			

^a^Anabolic Androgenic Steroids

^**b**^WADA prohibited substances from the following categories: Adrenergic and sympathomimetic agent; Diuretics, Sodium and potassium supplements; Hormones, Growth hormones, and hormone releasing agent; Post-cycle and adverse effects treatment agents and site-enhancing oils

**Fig. 3:**
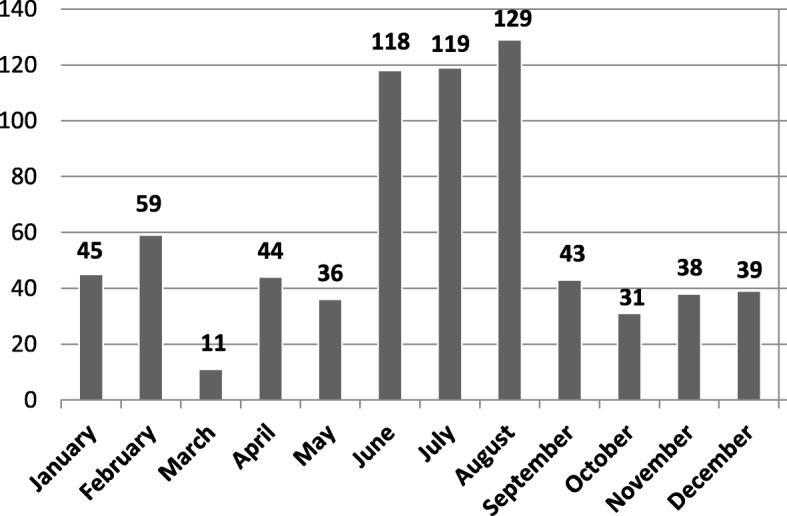
Number of seizures by months (2012–2017) (*N* = 712)

The most frequent month of seizures was August (*N* = 129), followed by July (*N* = 119), and June (*N* = 118), February (*N* = 59) and January (*N* = 45) as presented in Fig. 3.

## Discussion

As very little statistical analysis of law enforcement seizures of PES has been published to date [3, 17, 20], this research on seizures of performance-enhancing substances in Israel should provide an important addition to an area which merits further inquiry.

Israel represents an informative case of a region with extensive PES trafficking due to its relative economic and cultural isolation from its Arab neighbors. However, while most economic activity is carried out with countries that do not border Israel, counterfeit PES still arrives in considerable quantities from the Jordanian border. Even so, Israel PES markets exhibit diverse sources and procurement methods.

Our findings indicate that the source region from which PES seizures were most frequent was Eastern Europe, followed by East Asia, and West Asia. The country with the highest incident rate of seizures was Bulgaria, followed by Jordan, Thailand, and Moldova. Consistent with previous studies, the seized compounds were predominately manufactured in Asian countries, but probably dispatched to Western European nations via transit through South-Eastern Europe [29, 30, 33]. The higher number of seizures from Eastern Europe and Asia might be due to the former being a documented transit region. The latter region is the central production hub of AAS and doping substances [33].

Moreover, local law enforcement in some Eastern European countries is considered more permissive to PES trafficking. Moldova is a case in point, where AAS production is both legal and carried out by producers in major facilities [37]. Bulgaria, while not considered a large producer of anabolic steroids, is probably a transit state for AAS products and a frequent location of seizures [38, 39]. More importantly, it appears that local illicit distributors in Israel are connected with major suppliers of PES in Bulgaria, enacting supply networks by employing mail orders shipped to different shipping addresses. Nevertheless, Bulgaria had the highest rate of seized incidences, but the lowest quantity compared with other countries. This is explained by the fact that 69 out of the 71 (97.2%) seizures from Bulgaria were from mail packages, containing only a small numbers of items per seizure.

Jordan is a transit country for AAS, owing to their mostly unregulated status and widespread local use [33, 40]. Arab Gulf countries are supplied with doping substances through at least five channels: Trieste (Eastern Europe), the Turkey-Syria-Lebanon-Jordan route (Eastern Europe), India, China, and Switzerland. In sum, the routes of PES smuggling to Israel are diversified. Substances may arrive directly from producing countries, or by existing smuggling routes from both Europe and the Middle East.

Our findings also demonstrated significant regional difference related to gender, as most of the seizure cases involving women also involved PES of an Eastern European source. This could be due to the use of different addresses by local smugglers, some of which are registered to female tenants and property owners, as they presume this might incur a lower suspicion by enforcement agencies regarding package contents. The data also showed a marked difference in the incidence of seizures by years and place of seizures. Most of the PES seizures were from 2014, followed by 2016. This is probably due to shifting priorities within enforcement agencies, rather than an actual increase in illicit activity. In 2014, Israeli customs carried out seizures at both border crossings and Ben Gurion International Airport. Investigations of these seizures led to the identification of numerous clandestine labs. In contrast, similar efforts by customs were not evident in years prior, nor after 2014. Hence, 2014 was a distinctly active year for PES seizures by Israeli customs officials. As data indicates, the number of seizures is much higher at air and land points of entry. Moreover, all seizures reported to the Ministry of Health in the period analyzed, which were made at seaports, came from just two locations: Ashdod and Eilat. Conspicuously missing were seizures from Israel’s largest port - Haifa. We suspect this is due to the different priorities of Israeli customs within the Haifa port. Conversely, seizure of medicinal products was prominent in Ben-Gurion, as a great deal of resources, technology and manpower are employed to prevent entry of illicit substances to Israel. We suspect, that this indicates that rather from being centralized, seizure priorities of Israel customs vary greatly from area to area and from port to port. Additionally, it appears that there is need for further scrutiny of commercial Israeli sea-ports regarding their application of security measures for preventing the smuggling of PES.

Regional differences were also found regarding the incidence of seizures and place of seizures. Most airport seizures involved substances that arrived from East Asia, particularly Thailand, a known hub for the acquisition of PES. Seizures at the terrestrial border crossing from West Asia are overrepresented by the Kingdom of Jordan, as thousands of Arab-Israeli and Palestinians authority citizens cross this border every week. Seizures from central mail-processing centers represent the bulk of seizures from Eastern Europe Western Europe and East Asia. Thus, mail-package seizures represent the main avenue by which authorities confiscate illicit PES.

Also, regional differences were found regarding the type of substance seized. Adrenergic and sympathomimetic agents arrived mostly from Western Europe, while AAS mainly arrived from East Asia and Eastern Europe. The East Asia region, particularly countries like China [41], are known production sites for anabolic steroids, as well as Moldova and Ukraine.

The most common substance seized were AAS, consistent with previous reports [29, 42, 43]. Most seizures occurred during the summer season: August followed by July and then June. Because there is no similar seasonal information published from other countries, we can assume that in Israel, a small country with an extensive coastline as well as hot and sunny weather, demand for a sculpted body peaks in the summertime. This is increasingly plausible when we consider that AAS use appears to be more common among amateur athletes and bodybuilders in Israel - non-professional athletes. Thus, it can be concluded that PES use is a lifestyle choice [12, 15]. Moreover, it is evident that during summer, movement across country borders increases, as many people travel abroad for vacation.

Our results indicate that seizures were most common in the mail processing centers. These findings are consistent with related research carried out in other countries [3, 29, 30]. Relatively affordable products, accessible via the web [3], could explain the primacy of PES procurement through mail orders.

It is evident that PES use is a world-wide phenomenon, with various effects on health, sports and domestic medicine markets. No single jurisdiction is able to address the problem of PES trafficking and smuggling on its own. Hence, we suggest two overarching policies which could be adopted to better handle the issue of cross-border PES movements. First, we propose the adoption of a comprehensive plan on national anti-PES policy which will be formulated by representatives of multidisciplinary backgrounds such as law enforcement, education, sports, health and criminology. This could aid in tailoring enforcement measures to different populations. Second, we propose further cooperation using the current frameworks used by Interpol, the World Customs Organization, and the World Health Organization to integrate Israel into more robust networks of information exchange. These are successfully used when addressing drug control. Nevertheless, PES use is also a prominent health issue, and should be recognized with the appropriate salience and cooperation schemes available for drug control.

Two main possible interpretations of policy implications exist stemming from the results of this analysis. Firstly, as PES consistently enter Israel through various ports of entry, there is no reason to believe the phenomenon of PES trafficking will decrease or subside. The consistent numbers of seizures across the years of analysis exemplify this issue. Secondly, the effectiveness of enforcement efforts is currently limited to search and seizure operations, which do not result in investigation of perpetrators and traffickers. The fact that criminal investigations are seldom carried-out in seizure cases could be partly attributed to the shifting priorities of current responsible enforcement authorities, as trafficking in non-narcotic medicines is not considered a prime criminal activity. The ramifications of continued PES trafficking are not just increased risk to the health and well-being of users, but also tarnishing of competitive sport and the concept of fair-play.

Furthermore, trafficking of PES constitutes unrecorded money transactions which are not captured and taxed by the government. Nevertheless, PES trafficking and use is mainly a health issue. A possible policy response could be to transfer responsibility of enforcement in health-related issues to a dedicated Ministry of Health agency. The advantages of such a policy could result in more effective enforcement, but also in providing the needed expertise in drug analysis and drug regulation. Moreover, this could also provide additional tools for enforcement, such as imposing fines and economic sanctions against traffickers. These tools could be achieved by inserting new and specific provisions against PES in the current pharmacist ordinance or drafting a new dedicated PES law.

This study has several limitations. First, our sample only represents the substances that were found by enforcement authorities and may not fully reflect the phenomenon. Second, this study is based on case files with limited information on operational details, and some of the details such as country of origin, gender, age and the identity of suspects are either unknown or have not been validated. Third, not all the substances have been confirmed in lab analysis and could represent substances other than PES or different PES other than those reported. The parity between reported cases and analytically confirmed substances is mainly due to necessity and budgetary restrictions. Fifth, a single pharmacist selected the cases, according to inclusion criteria and another provided quality control. Nevertheless, this process could have led to selection bias, with omission of other relevant cases or inclusion of irrelevant ones.

Until 2016, criminal proceedings necessitated only the identification of an illicit PES by a pharmacist, reserving analytical confirmation only to cases involving unlabeled products or raw powder. Moreover, budget restrictions limited the number of products, which could be sent to a lab for analysis. Lastly, we could not determine real significance of differences between regions as not all the requirements of chi-square tests were met (Expected counts< 5).

## Conclusions

The data analysis in this study provides new insights into the Israeli illegal PES market. This knowledge can be further used to improve our understanding of local as well as international markets. This study also demonstrates that Israel is a thriving hub for the importation and distribution of PES. The trends uncovered in this study emphasize the need for heightened enforcement activity at Israel’s borders and in mail processing centers. Particular attention should be given to merchandise arriving from Eastern Europe, East Asia, and the Israeli-Jordan border, particularly in the summertime.

WADA is increasingly shifting emphasis from athlete testing to cooperation and intelligence sharing with local law enforcement, defined as activities shaping “the future of anti-doping” [3].

Unfortunately, not all information on police and customs cases involving PEDs is passed to the DEI, and much of what is already provided is at the discretion of both agencies. Some information, particularly pertaining to ongoing investigations is purposely withheld due to privacy or confidentiality considerations. Evidently, better coordination is required by all agencies in information sharing, to optimally enforce the ban on illicit PEDs.

In Israel, policymakers should consider not just prioritizing law enforcement action and intelligence sharing, but also reforming the penal law to criminalize PES trafficking. This has already been done in countries like Spain, Italy, and Belgium. Reforms will likely incentivize the enforcement activities of law-enforcement agencies. Furthermore, policy-makers should consider enhancing health ministry agencies with higher enforcement capacity by giving them further investigative and inquiry authority. Lastly, more studies should be done regarding the nature of the PES market in Israel, from criminological, sociological, and ethnographic perspectives in order to further our holistic understanding. The data analysis focused on the supply side of the PES market, although due to the many seizures of performance-enhancing substances, it can be assumed that this market has a wide consumer base and growing demand. Hence developing a comprehensive PES policy in Israel involving prevention, enforcement and health promotion measures that are not currently applied in Israel is imperative.

## Data Availability

The data used in this paper is based on pharmaceutical crime cases recorded in the DEI electronic database.

## Abbreviations

AAS: Anabolic-androgenic steroids
DEI: Division of Enforcement and Inspection
IADA: Israel Anti-Drug Authority
IADAV: Israel Anti-Drug, Alcohol and Violence Authority
INADO: Israel National Anti-Doping Organization
PES: Performance-enhancing substances
PIED: Performance and image enhancing drugs
UNESCO: United Nations Educational, Scientific and Cultural Organization
WADA: World Anti-Doping Agency
